# Body Snatching—Resurrectionists—The Remedy

**Published:** 1857-11

**Authors:** 


*Body Snatching—Resurrectionists—The Remedy.*

Many of our readers will see from paragraphs in the newspapers, copied from those of this city, accounts of the arrest of the City Sexton and one of the students attending the College, on a charge of having been engaged in taking bodies from the cemetery for purposes of dissection. The student easily proved that he had nothing to do with the affair, and was discharged; the sexton was held to bail. As a proper supply of subjects for dissection, in all our medical colleges, is a matter of much importance, both to the profession and the community, we copy the following letter addressed to the editors of one of our daily papers, and hope that some systematic effort may be made to remedy the evil complained of:

EDITORS OF THE CHICAGO DAILY TRIBUNE:

In a recent number of your paper you took occasion to comment with severity upon the practice of obtaining *bodies* for dissection, and expressed your regret that the Legislature had not made the alleged crime punishable by imprisonment in the penitentiary.

While the public attention is turned to this subject, I wish to make a few suggestions concerning it, and see if there is not a better remedy than the one you propose.

It is easy to talk flippantly about the sanctity of the grave, and to increase popular prejudice by denouncing every disturbance of the dead as the work of "hyenas" and *barbarians.* But it is one of the plainest principles of law, that the criminality of an act or its non-criminality must be determined by the motives that induced its committal, and its effect upon the life, property, or health of others. For any citizen to commit an act which is directly calculated to injure the health or property of another is plainly criminal, even if the motive which impelled him to its committal was actual hunger or extreme destitution. The criminality would consist in the trespass upon the rights of others. Again, if a citizen should commit an act which disregarded the moral sense and peace of the community, for no useful purpose, such as opening a grave and leaving the body exposed and mutilated, it would be highly criminal; but if the body was obtained as carefully and with as little disturbance of the public peace as possible, and used *solely* for the purpose of gaining such knowledge as would be of essential benefit to the living, it would require a pretty active imagination to recognize in such an act either criminality or injustice. Certainly, the *motive* cannot be impeached; and are the rights or property of any one invaded by the taking of the body? When a human body is deposited among the undistinguished and undistinguishable dead of the potter's field, there to decay or be eaten up by worms, it is as absurd to claim that it remains the "property" of any one, as it would be for the previous owner to re-claim the right of property in the old boots that he had cast off and thrown into the streets.

If, then, dissections of the human body are absolutely necessary to qualify the physician and surgeon to discharge their duties to the living, the procurement of material for such necessary purpose cannot be *in its own nature* criminal. It is true that our legislators have enacted laws declaring such act criminal, and provided heavy penalties to deter from its commission. But the same legislators have also passed laws by which every physician and surgeon is made liable to suits and heavy damages for mal-practice, if they do not daily use—and use skilfully, too—the very knowledge which can be gained in no other way than by dissections of the human body. Thus, legally, the medical profession are placed very much in the condition of the Israelites in Egypt, when they were required to make the full number of bricks daily, but denied the straw or materials necessary to make them of. The absurdity and injustice of keeping a whole profession in such a position in relation to the laws must be obvious to every reasonable citizen.

Now, Mr. Editor, before you publish anything further concerning "hyenas" and barbarians in connection with the procurement of subjects solely for dissection, please answer honestly the following questions: If you should be unfortunate enough to get your leg crushed, perhaps, in one of the many railroad accidents, so as to require amputation, and a *professional* surgeon was called who had rigidly obeyed the laws of the State by abstaining from dissection; having never traced the exact position of the arteries, he, in cutting off the limb, either lets you bleed to death or arrests the flow of blood by searing the whole stump with a red-hot iron, as all surgeons did before dissections were known—which would you think most deserving of the penitentiary, such a pretended surgeon, or one who, by the careful and patient dissection of a dozen dead bodies, had learned the exact position of every vessel, so as to amputate a limb without the slightest danger from loss of blood? Or, if you had an *aneurism*, a disease of a large artery, which, if let alone, would inevitably be fatal, would you prefer a surgeon who had never dissected an artery in his life, or one who, having dissected minutely, knew exactly where to cut down, and with a thread tie up the diseased vessel and save your life? Or, again, if the one you love more than all others, while lying upon her couch after having given birth to your first born, was attacked with a sudden and most dangerous flow of blood, would you want a physician by her side whose minute knowledge of anatomy would enable him to place his finger on the main artery and arrest the wasting tide of life in a moment, or one of those *good souls* whose regard for the sanctity of the dead had left him in entire ignorance of the situation of the artery or of any means of immediate relief?

Let all those good citizens who are disposed to hold up their hands in holy horror whenever a coffin in the potter's field is found empty, bring these questions home to themselves. But you are ready to ask if I openly advocate the robbing of graveyards. I answer, no! I do advocate, however, such a change in our laws as will render the robbing of graveyards unnecessary. Just as long as justice to the living absolutely requires of the physician knowledge which he can get in no other way than by dissecting dead bodies, and the laws hold him responsible for the actual use of such knowledge in the treatment of his patients, so long graveyards will be robbed, no matter what the penalty may be, unless the laws make some adequate provision for suplying a sufficient number without robbing.

Now, Mr. Editor, if you and the rest of our good citizens, instead of asking the next Legislature to make the taking of bodies from the potter's field a *penitentiary* offence, will unite in asking for and obtaining the passage of a law, like the one already enacted in New York, by which those who die in our hospitals, poor-houses, jails, penitentiaries, etc., and are not claimed for burial by friends within a specified time, shall be given up for dissection under proper regulations, there will be no further disturbance in the burial places for the dead. The exhuming of dead bodies is not so pleasant a task, that men will engage in it for mere sport, or without a strong necessity. Only give to the profession a reasonable supply to enable its members to do justice to the living by the enactment of consistent laws, and you may be assured that every dead body, which has friends who care enough about it to claim it for burial, will be allowed to rest peacefully in its mother earth.

